# MAGA Republicans’ views of American democracy and society and support for political violence in the United States: Findings from a nationwide population-representative survey

**DOI:** 10.1371/journal.pone.0295747

**Published:** 2024-01-03

**Authors:** Garen J. Wintemute, Sonia L. Robinson, Elizabeth A. Tomsich, Daniel J. Tancredi

**Affiliations:** 1 Department of Emergency Medicine, Violence Prevention Research Program, and California Firearm Violence Research Center, University of California, Davis, Sacramento, California, United States of America; 2 Department of Pediatrics, University of California, Davis, Sacramento, California, United States of America; St John’s University, UNITED STATES

## Abstract

**Background:**

Identifying groups at increased risk for political violence can support prevention efforts. We determine whether “Make America Great Again” (MAGA) Republicans, as defined, are potentially such a group.

**Methods:**

Nationwide survey conducted May 13-June 2, 2022 of adult members of the Ipsos KnowledgePanel. MAGA Republicans are defined as Republicans who voted for Donald Trump in the 2020 presidential election and deny the results of that election. Principal outcomes are weighted proportions of respondents who endorse political violence, are willing to engage in it, and consider it likely to occur.

**Findings:**

The analytic sample (n = 7,255) included 1,128 (15.0%) MAGA Republicans, 640 (8.3%) strong Republicans, 1,571 (21.3%) other Republicans, and 3,916 (55.3%) non-Republicans. MAGA Republicans were substantially more likely than others to agree strongly/very strongly that “in the next few years, there will be civil war in the United States” (MAGA Republicans, 30.3%, 95% CI 27.2%, 33.4%; strong Republicans, 7.5%, 95% CI 5.1%, 9.9%; other Republicans, 10.8%, 95% CI 9.0%, 12.6%; non-Republicans, 11.2%, 95% CI 10.0%, 12.3%; p < 0.001) and to consider violence usually/always justified to advance at least 1 of 17 specific political objectives (MAGA Republicans, 58.2%, 95% CI 55.0%, 61.4%; strong Republicans, 38.3%, 95% CI 34.2%, 42.4%; other Republicans, 31.5%, 95% CI 28.9%, 34.0%; non-Republicans, 25.1%, 95% CI 23.6%, 26.7%; p < 0.001). They were not more willing to engage personally in political violence.

**Interpretation:**

MAGA Republicans, as defined, are more likely than others to endorse political violence. They are not more willing to engage in such violence themselves; their endorsement may increase the risk that it will occur.

## Introduction

A growing body of research [1–6], assessments by experts in domestic violent extremism [7], and events such as the insurrection of January 6, 2021 have raised concern about the prospect of widespread political violence in the United States (US). Political violence, like other forms of violence, kills and injures people; even the credible possibility of political violence has negative health and social consequences at the individual and population levels. Political violence should be studied and addressed as a public health problem.

In late spring 2022 we conducted the Life in America Survey to develop a better understanding of political violence based on data from a large nationally representative US sample. The survey’s first report found concerning levels of support for political violence—including personal willingness to engage in that violence [6]. Because understanding the distribution of risk for adverse health events is fundamental to the public health approach, additional reports have focused on variation in support for political violence associated with political party affiliation and political ideology [8] and with firearm ownership [9].

This report focuses on one specific group that may be at increased risk for political violence: so-called MAGA (Make America Great Again) Republicans. In speeches on August 25, 2022, in Bethesda and Rockville, Maryland [10, 11], and September 1, 2022, in Philadelphia, Pennsylvania [12], US President Biden used that term in reference to Republicans who supported Donald Trump and denied the results of the 2020 election. He asserted that MAGA Republicans endorsed political violence, implying that they did so more than others or that others did not. He emphasized his belief that his characterization applied to only a minority of Republicans [13–16]. Critics nonetheless accused him of maligning half the country, apparently referring to persons who had voted for Donald Trump [13, 17–20]. These critics were asserting, in essence, that MAGA Republicans were indistinguishable from other Republicans.

These events generated the two research questions that we explore in this analysis. To begin, and in accordance with the history described above, we define MAGA Republicans as Republicans who voted for Donald Trump in 2020 and agreed strongly or very strongly with the statement that “the 2020 election was stolen from Donald Trump, and Joe Biden is an illegitimate president.” We establish three comparison groups: self-identified strong Republicans, other Republicans, and non-Republicans.

The first research question is, Are MAGA Republicans a distinct subset of Republican party affiliates, other than on the characteristics incorporated in their definition? We compare MAGA Republicans with the other groups on demographic characteristics and an array of beliefs about American democracy and society. The second question is the primary focus of this analysis: Are MAGA Republicans more likely than others to see political violence as justified and to be willing to engage personally in political violence? We compare them with others on the beliefs that political violence is justified generally and to advance 17 specific political objectives. We also assess their relative willingness to engage in political violence at varying levels of lethality and against nine specific classes of people, defined by occupational or social characteristics. Finally, we compare MAGA Republicans with others on their willingness to possess and use firearms in situations where they consider political violence to be justified.

## Methods

The 2022 Life in America Survey was designed by the authors and administered online in English and Spanish from May 13 to June 2, 2022, by the survey research firm Ipsos [21]. The survey’s methods have been described in detail elsewhere [6, 8] and are summarized here. The study is reported following American Association for Public Opinion Research guidelines [22].

### Ethics statement

This study protocol was reviewed by the University of California Davis Institutional Review Board (UC Davis IRB ID 187125–1) and determined to be exempt (category 2: survey research) from full committee review. The University of California, Davis, in accordance with its Federalwide Assurance (FWA) for the Protection of Human Subjects agreement with the Department of Health & Human Services (FWA 00004557), adheres to all federal and state regulations related to the protection of human research subjects, including 45 CFR 46 (“The Common Rule”), 21 CFR 50 and 21 CFR 56 for FDA regulated products, and the principles of The Belmont Report and Institutional policies and procedures. In addition, the International Conference on Harmonization, Good Clinical Practice (ICH GCP) principles are adhered to insofar as they parallel the previously mentioned regulations and policies.

### Consent to participate

The UC Davis IRB waived a requirement for written or verbal consent. Instead, introductory text to the questionnaire as seen by participants included this statement:

As with all KnowledgePanel surveys, your participation is entirely voluntary, and your responses will be kept confidential and anonymous. You will not be individually identified, and your de-identified responses will only be used for qualified research purposes. You may skip any question at any time.

If you have any questions about this survey, you may contact the research team by calling (916) 734–3539. This study has been approved by the Institutional Review Board of the University of California, Davis. If you have any questions about your rights as a participant in this study, you may contact the University of California, Davis, Institutional Review Board at (916) 703–9151. If you have questions about your rights as a research subject or are dissatisfied at any time with any aspect of the survey, you may also contact KnowledgePanel panel member support at (800) 782–6899.

By continuing, you are agreeing to participate in this study.

### Participants

Respondents were drawn from the Ipsos KnowledgePanel, an online research panel that has been widely used in population-based research, including studies of violence and firearm ownership [23–25].

To establish a nationally representative panel, members are recruited on an ongoing basis through address-based probability sampling using data from the US Postal Service’s Delivery Sequence File, a method that facilitates adequate representation from hard-to-reach populations [26, 27]. Recruitment into KnowledgePanel involves repeated contact attempts, if necessary, by mail and telephone. Recruited adults in households without internet access are provided a web-enabled device and free internet service, and a modest, primarily points-based incentive program seeks to encourage participation and promote participants’ retention in KnowledgePanel over time.

A probability-proportional-to-size procedure was used to select a study-specific representative sample of KnowledgePanel members. All panel members who were aged 18 years and older were eligible for selection. Invitations were sent by e-mail; automatic reminders were delivered to non-respondents by e-mail and telephone beginning 3 days later.

A final survey weight variable provided by Ipsos adjusted for the initial probability of selection into KnowledgePanel and for survey-specific nonresponse and over- or under-coverage using design weights with post-stratification raking ratio adjustments. With weighting, the sample is designed to be statistically representative of the noninstitutionalized adult population of the US as reflected in the 2021 March supplement of the Current Population Survey [26, 27].

### Measures

Sociodemographic data, political party affiliation, and voting behavior were collected by Ipsos from profiles created and maintained by KnowledgePanel members. Party affiliation is derived following a procedure used since the 1950s by the American National Election Studies (ANES) [28]. As described in the report of a previous KnowledgePanel survey on another topic,

Respondents were first asked, “Generally speaking, do you think of yourself as” (response choices: “Republican,” “Democrat,” “Independent,” “Another party,” “No preference”). Republicans/Democrats were then asked, “Would you call yourself a” (response choices: “Strong Republican/Democrat,” “Not very strong Republican/Democrat”). All others were asked the follow-up question: “Do you think of yourself as closer to the” (response choices: “Republican Party,” “Democratic Party”)

[29].

Respondents who answered this last question were coded by Ipsos as “Leans Republican” or “Leans Democrat.” Respondents who refused to answer (n = 165, 2.7% of the sample) were coded as “Undecided/Independent/Other.” The Democrat, Leans Democrat, and Undecided/Independent/Other groups were combined for analysis as non-Republicans.

Survey questions that supplied data for this analysis covered three broad domains: beliefs regarding democracy and the potential for violence in the US; beliefs regarding American society and institutions; and support for and personal willingness to engage in violence, including political violence. Prior surveys on these topics were reviewed, and selected questions were included or adapted in this questionnaire to track trends in opinion and provide context for responses to questions that had not been asked previously.

Support for the QAnon delusion complex was determined through respondents’ agreement with statements of core elements of that complex: that US institutions “are controlled by a group of Satan-worshipping pedophiles who run a global child sex trafficking operation” and that “a storm coming soon” will “sweep away the elites in power and restore the rightful leaders.” Support for great replacement thinking was determined through respondents’ agreement with the statement that “in America, native-born white people are being replaced by immigrants.”

Our primary outcome measures concerned political and non-political violence. Violence was represented by the phrase “force or violence,” defined as “physical force strong enough that it could cause pain or injury to a person.” “Force or violence to advance an important political objective that you support” was used in questions about respondents’ support for and willingness to engage in political violence.

Respondents were asked about the extent to which they considered political violence to be justified “in general” and then about justification for its use to advance specified political objectives, such as “to return Donald Trump to the presidency this year,” “to preserve an American way of life based on Western European traditions,” and “to stop police violence.” There were 17 specified objectives. Nine were presented to all respondents, and 8 were paired, with each respondent seeing only 1 item from each pair; each respondent was presented with 13 of 17 objectives.

Respondents who considered political violence to be at least sometimes justified for at least 1 of these specified objectives were asked about their personal willingness to engage in political violence: by type of violence (to “damage property,” “threaten or intimidate a person,” “injure a person,” “kill a person”) and by target population (examples: “an elected federal or state government official,” “a police officer,” “a person who does not share your religion”).

All respondents were asked about the likelihood of their future use of firearms in a situation where they consider political violence to be justified (e.g., “I will be armed with a gun”; “I will shoot someone with a gun”).

The full text of all survey questions reported on here is in the Supplement.

### Implementation

Forty KnowledgePanel members participated in a pretest of the English language version that was administered April 27 to May 2, 2022.

Respondents were randomized 1:1 to receive response options in order from negative to positive valence (e.g., from ‘do not agree’ to ‘strongly agree’) or the reverse throughout the questionnaire. Where a question presented multiple statements for respondents to consider, the order in which those statements were presented was randomized unless ordering was necessary. The questionnaire included several design elements intended to minimize inattentive responses and biased estimation of support for political violence [6].

We employed unipolar response arrays without a neutral midpoint (e.g., do not agree, somewhat agree, strongly agree, very strongly agree). The literature is not in agreement on whether such midpoints should be included [5, 30]. We were persuaded by the studies reviewed by Chyung et al. [30], which suggest that such midpoints facilitate selection of “a minimally acceptable response as soon as it is found,” known as satisficing. According to those authors, satisficing is particularly common when respondents are uncomfortable with the topics of the survey or under social desirability pressures, and both conditions apply here. In the analysis, we focus on responses above the “somewhat” or “sometimes” level to minimize the impact of potential satisficing on the results.

The questionnaire included a check for inattentiveness among respondents: two fictitious entries were included in lists of news and information outlets for which respondents provided information on their extent of use.

### Statistical analysis

In forming the analytic sample for this study, we excluded the 1,365 of 8,620 respondents for whom party affiliation, 2020 voting behavior, or opinion on the legitimacy of the 2020 presidential election was not available. To generate prevalence estimates, we calculated weighted percentages and 95% confidence intervals (CI) using PROC SURVEYFREQ and weighted averages with standard errors in PROC SURVEYMEANS in SAS version 9.4 (SAS Institute, Inc., Cary, NC). Outcomes were defined dichotomously to produce prevalence differences. Unadjusted and adjusted prevalence differences and their 95% confidence intervals (CI) were determined by PROC SURVEYREG, with robust standard errors to correct for design effects and heteroskedasticity in binary outcomes. Two multivariable models were used to adjust for covariates: Model 1 included age, gender, and race and ethnicity; Model 2 retained those variables and added income, education, and census division. P-values were corrected for multiple comparisons by controlling the false discovery rate using the Benjamini-Hochberg method [31]. The article text presents the absolute percentages and design-adjusted F-test p-values from Model 2. Tables with prevalence differences for the unadjusted models, Model 1, and Model 2 are presented in the Supplement.

Estimated counts of adults in the US were generated by simple extrapolation, multiplying weighted percentages and their confidence intervals from our sample by the estimated adult population of the US as of July 1, 2021 (258.33 million persons) [32].

A sensitivity analysis excluded the 447 respondents who reported use of one or both fictitious news sources.

## Results

Of 15,449 panel members invited to participate, 8,620 completed the survey, yielding a 55.8% completion rate. Information on the demographics of respondents and nonrespondents is provided elsewhere [6].

The 7,255 respondents who made up the analytic sample for this study comprised 1,128 MAGA Republicans (15.0%, 95% CI 14.1%, 15.9%), 640 strong Republicans (8.3%, 95% CI 7.6%, 9.0%), 1,571 other Republicans (21.3%, 95% CI 20.3%, 22.4%), and 3,916 non-Republicans (55.3%, 95% CI 54.1%, 56.6%). We estimate that MAGA Republicans, as defined, account for 33.6% (95% CI 31.9%, 35.4%) of all Republicans and15.0% (95% CI 14.1%, 15.9%) of the adult population of the US, or approximately 38.8 million (95% CI 36.5 million, 41.1 million) people.

### Demographic characteristics

Among the three groups of Republicans, age and race and ethnicity distributions were similar; MAGA Republicans were more likely to be female (Table 1). MAGA Republicans were less likely than all other groups to have a bachelor’s or postgraduate degree or a household income of $150,000 or greater.

**Table 1:** Demographic and socioeconomic characteristics of study groups.

Characteristic	Republicans						Non-Republicans	
	MAGA Republicans*		Strong Republicans		Other Republicans			
	Unweighted n	Weighted % (95% CI)	Unweighted n	Weighted % (95% CI)	Unweighted n	Weighted % (95% CI)	Unweighted n	Weighted % (95% CI)
**Overall**	1128	15.0 (14.1, 15.9)	640	8.3 (7.6, 9.0)	1571	21.3 (20.3, 22.4)	3916	55.3 (54.1, 56.6)
**Age**								
18–24	36	6.4 (4.2, 8.7)	21	7.2 (4.2, 10.2)	66	8.1 (6.1, 10.1)	191	10.1 (8.7, 11.5)
25–34	75	9.4 (7.3, 11.6)	50	11.3 (8.2, 14.4)	165	15.2 (12.9, 17.5)	526	18.2 (16.7, 19.7)
35–44	165	17.7 (15.1, 20.2)	74	13.7 (10.7, 16.7)	249	18.3 (16.1, 20.4)	612	18.3 (16.9, 19.7)
45–54	155	15.1 (12.8, 17.4)	81	13.9 (11.0, 16.8)	260	16.8 (14.9, 18.8)	477	12.5 (11.4, 13.7)
55–64	266	21.0 (18.5, 23.4)	162	23.2 (19.8, 26.6)	354	19.4 (17.4, 21.4)	813	17.1 (15.9, 18.3)
65–74	282	19.7 (17.4, 22.0)	158	19.3 (16.3, 22.2)	292	13.3 (11.7, 14.8)	868	15.5 (14.4, 16.6)
75+	149	10.7 (8.9, 12.5)	94	11.4 (9.1, 13.7)	185	8.9 (7.5, 10.2)	429	8.2 (7.3, 9.0)
**Gender**								
Female	565	52.4 (49.2, 55.6)	284	43.9 (39.7, 48.1)	669	43.8 (41.0, 46.5)	2059	52.9 (51.1, 54.6)
Male	552	47.4 (44.2, 50.7)	346	55.1 (50.9, 59.3)	880	55.6 (52.9, 58.4)	1784	45.0 (43.3, 46.8)
Other	2	0.2 (0.0, 0.4)	6	1.1 (0.2, 1.9)	11	0.6 (0.2, 1.0)	63	2.1 (1.6, 2.7)
**Race/Ethnicity**								
White, Non-Hispanic	942	81.0 (78.1, 84.0)	570	87.7 (84.8, 90.7)	1281	77.0 (74.4, 79.7)	2486	55.4 (53.6, 57.2)
Black, Non-Hispanic	18	2.1 (0.9, 3.2)	9	2.1 (0.7, 3.5)	55	4.5 (3.2, 5.9)	614	19.1 (17.6, 20.6)
Hispanic, any race	88	9.3 (7.3, 11.3)	41	7.3 (5.0, 9.6)	118	10.2 (8.3, 12.1)	499	16.9 (15.5, 18.4)
American Indian or Alaska Native, Non-Hispanic	8	1.6 (0.4, 2.8)	3	0.5 (0.0, 1.0)	12	1.6 (0.6, 2.5)	17	0.8 (0.4, 1.2)
Asian American / Pacific Islander, non-Hispanic	23	3.9 (2.1, 5.8)	9	1.9 (0.6, 3.2)	50	4.8 (3.4, 6.2)	154	5.6 (4.7, 6.6)
Some other race, Non-Hispanic	2	0.2 (0.0, 0.4)	0	0 (N/A)	5	0.2 (0.0, 0.3)	13	0.2 (0.1, 0.3)
2+ Races, Non-Hispanic	47	1.9 (1.3, 2.5)	8	0.6 (0.1, 1.1)	50	1.8 (1.1, 2.4)	133	2.0 (1.5, 2.5)
**Marital status**								
Now married	770	66.7 (63.5, 69.9)	465	70.2 (66.2, 74.2)	1019	61.0 (58.3, 63.8)	2245	52.4 (50.7, 54.2)
Widowed	67	5.3 (4.0, 6.7)	33	3.9 (2.5, 5.2)	74	3.5 (2.7, 4.4)	215	4.3 (3.7, 4.9)
Divorced or Separated	142	11.1 (9.2, 13.0)	64	9.7 (7.3, 12.1)	201	11.8 (10.1, 13.4)	472	9.9 (9.0, 10.9)
Never married	149	16.8 (14.0, 19.7)	78	16.3 (12.7, 19.8)	277	23.7 (21.0, 26.3)	984	33.3 (31.5, 35.1)
**Education**								
No high school diploma or GED	52	5.9 (4.2, 7.7)	19	4.8 (2.5, 7.0)	70	6.3 (4.7, 7.8)	181	7.0 (5.9, 8.1)
High school graduate (diploma, GED)	396	38.4 (35.2, 41.6)	184	31.8 (27.7, 35.8)	351	24.7 (22.2, 27.1)	804	23.9 (22.3, 25.5)
Some college or Associate’s degree	382	33.5 (30.4, 36.5)	187	29.7 (25.9, 33.6)	442	29.0 (26.5, 31.5)	1025	26.2 (24.7, 27.8)
Bachelor’s degree	182	13.8 (11.8, 15.8)	157	21.0 (17.8, 24.2)	407	23.3 (21.1, 25.5)	990	23.0 (21.6, 24.4)
Master’s degree or higher	116	8.4 (6.8, 10.0)	93	12.8 (10.1, 15.4)	301	16.8 (14.9, 18.7)	916	19.9 (18.6, 21.2)
**Household Income**								
Less than $10,000	23	2.2 (1.2, 3.1)	10	2.6 (0.9, 4.2)	36	2.7 (1.8, 3.7)	119	3.7 (3.0, 4.5)
$10,000 to $24,999	93	8.2 (6.5, 10.0)	35	5.9 (3.8, 7.9)	100	6.6 (5.2, 8.0)	335	8.6 (7.6, 9.6)
$25,000 to $49,999	234	19.3 (16.9, 21.8)	104	15.3 (12.4, 18.2)	225	13.4 (11.5, 15.2)	601	15.2 (13.9, 16.5)
$50,000 to $74,999	196	18.1 (15.5, 20.8)	116	17.0 (13.9, 20.1)	251	15.6 (13.6, 17.6)	628	16.1 (14.8, 17.5)
$75,000 to $99,999	163	13.1 (11.0, 15.1)	85	12.6 (9.9, 15.4)	234	14.8 (12.9, 16.8)	562	13.3 (12.1, 14.4)
$100,000 to $149,999	205	20.1 (17.4, 22.7)	120	20.3 (16.8, 23.8)	286	19.1 (16.9, 21.4)	705	18.4 (17.0, 19.8)
$150,000 or more	214	19.0 (16.5, 21.5)	170	26.4 (22.7, 30.1)	439	27.7 (25.3, 30.1)	966	24.7 (23.2, 26.2)
**Employment**								
Working full time	451	44.8 (41.5, 48.0)	290	48.8 (44.6, 53.0)	786	52.9 (50.1, 55.6)	1774	49.1 (47.3, 50.9)
Working part time	129	11.1 (9.1, 13.2)	75	13.4 (10.3, 16.6)	204	13.6 (11.7, 15.6)	528	14.8 (13.5, 16.1)
Not working—retired	414	29.8 (27.1, 32.5)	228	28.2 (24.7, 31.6)	428	21.0 (19.0, 23.0)	1153	21.5 (20.3, 22.8)
Not working—not retired	134	14.3 (11.9, 16.7)	47	9.6 (6.8, 12.5)	153	12.5 (10.5, 14.5)	461	14.6 (13.3, 16.0)
**Census division**								
New England	49	4.1 (2.8, 5.3)	20	2.9 (1.6, 4.2)	82	5.3 (4.1, 6.6)	213	5.5 (4.7, 6.3)
Mid-Atlantic	136	11.3 (9.3, 13.4)	84	13.0 (10.2, 15.8)	206	13.0 (11.2, 14.8)	513	13.4 (12.1, 14.6)
East-North Central	158	13.8 (11.7, 15.9)	102	16.3 (13.1, 19.4)	244	14.9 (13.0, 16.8)	581	14.5 (13.3, 15.8)
West-North Central	90	8.0 (6.3, 9.8)	51	7.9 (5.6, 10.1)	120	7.3 (6.0, 8.7)	262	5.9 (5.2, 6.7)
South Atlantic	237	21.8 (19.1, 24.5)	132	19.3 (16.1, 22.5)	292	19.0 (16.9, 21.1)	768	20.4 (19.0, 21.9)
East-South Central	81	8.7 (6.7, 10.7)	52	9.1 (6.6, 11.7)	74	5.1 (3.8, 6.4)	179	4.8 (4.0, 5.6)
West-South Central	136	12.4 (10.2, 14.6)	79	14.3 (11.1, 17.5)	175	13.0 (11.1, 15.0)	349	10.2 (9.1, 11.4)
Mountain	107	8.5 (6.8, 10.2)	60	8.2 (6.1, 10.3)	150	8.2 (6.8, 9.5)	312	7.0 (6.2, 7.9)
Pacific	134	11.3 (9.3, 13.4)	60	9.1 (6.6, 11.5)	228	14.1 (12.2, 16.1)	739	18.1 (16.8, 19.5)

* Denotes self-identified Republicans who voted for Donald Trump in 2020 and agreed strongly or very strongly with the statement that “the 2020 election was stolen from Donald Trump, and Joe Biden is an illegitimate president.”

### Democracy, the potential need for violence, and civil war

MAGA Republicans were more likely than others to think that “there is a serious threat to our democracy” (MAGA Republicans, 90.0%, 95% CI 87.9%, 92.0%; strong Republicans, 74.4%, 95% CI 70.6%, 78.1%; other Republicans, 61.7%, 95% CI 59.0%, 64.4%; non-Republicans, 70.1%, 95% CI 68.4%, 71.8%; p< 0.001) (Table 2, S1 Table in S1 File). They were more likely to agree strongly or very strongly that “having a strong leader for America is more important than having a democracy” (MAGA Republicans, 31.0%, 95% CI 28.0%, 34.1%; strong Republicans, 17.8%, 95% CI 14.5%, 21.2%; other Republicans, 17.0%, 95% CI 14.8%, 19.2%; non-Republicans, 15.2%, 95% CI 13.9%, 16.6%; p< 0.001) and that “armed citizens should patrol polling places at election time” (MAGA Republicans, 19.2%, 95% CI 16.6%, 21.9%; strong Republicans, 5.0%, 95% CI 2.8%, 7.1%; other Republicans, 4.1%, 95% CI 2.9%, 5.4%; non-Republicans, 3.6%, 95% CI 2.9%, 4.3%; p< 0.001).

**Table 2:** Beliefs concerning democracy in the US, by study group.

Statement	Republicans						Non-Republicans	
	MAGA Republicans*		Strong Republicans		Other Republicans			
	Unweighted n	Weighted % (95% CI)	Unweighted n	Weighted % (95% CI)	Unweighted n	Weighted % (95% CI)	Unweighted n	Weighted % (95% CI)
Do you believe that things in this country today are…								
Generally headed in the wrong direction	1110	98.7 (98.0, 99.4)	608	95.6 (93.7, 97.4)	1419	89.7 (88.0, 91.4)	2874	74.4 (72.9, 76.0)
Generally headed in the right direction	15	1.3 (0.6, 2.0)	26	4.4 (2.6, 6.3)	147	10.3 (8.6, 12.0)	1011	25.6 (24.0, 27.1)
When thinking about democracy in the United States these days, do you believe…								
There is a serious threat to our democracy.	1025	90.0 (87.9, 92.0)	480	74.4 (70.6, 78.1)	993	61.7 (59.0, 64.4)	2859	70.1 (68.4, 71.8)
There may be a threat to our democracy, but it is not serious.	77	8.1 (6.1, 10.0)	118	19.3 (15.9, 22.7)	451	30.2 (27.6, 32.7)	812	23.2 (21.6, 24.8)
There is no threat to our democracy.	23	2.0 (1.1, 2.8)	40	6.3 (4.2, 8.4)	121	8.1 (6.6, 9.7)	224	6.7 (5.8, 7.7)
How important do you think it is for the United States to remain a democracy?								
Not important	24	2.5 (1.4, 3.5)	9	1.8 (0.5, 3.0)	23	1.9 (1.0, 2.7)	47	1.5 (1.0, 2.0)
Somewhat important	44	4.4 (3.0, 5.8)	23	4.8 (2.7, 6.9)	85	6.7 (5.2, 8.3)	190	6.6 (5.6, 7.6)
Very or extremely important	1052	93.1 (91.4, 94.8)	607	93.4 (91.0, 95.8)	1459	91.4 (89.7, 93.1)	3667	91.9 (90.8, 93.0)
Democracy is the best form of government.								
Do not agree	94	9.2 (7.2, 11.2)	23	4.5 (2.6, 6.5)	74	5.6 (4.3, 7.0)	120	3.9 (3.2, 4.7)
Somewhat agree	170	16.1 (13.7, 18.5)	90	16.3 (13.0, 19.6)	301	21.2 (18.8, 23.5)	829	25.1 (23.5, 26.7)
Strongly or very strongly agree	856	74.7 (71.8, 77.6)	524	79.1 (75.5, 82.8)	1193	73.2 (70.7, 75.8)	2948	71.0 (69.3, 72.7)
These days, American democracy only serves the interests of the wealthy and powerful.								
Do not agree	378	31.5 (28.5, 34.4)	343	50.8 (46.6, 55.0)	588	33.3 (30.8, 35.8)	934	21.2 (19.8, 22.6)
Somewhat agree	293	27.0 (24.1, 29.9)	184	30.6 (26.7, 34.6)	553	36.9 (34.2, 39.6)	1505	38.1 (36.4, 39.8)
Strongly or very strongly agree	446	41.6 (38.3, 44.8)	112	18.6 (15.2, 22.0)	428	29.8 (27.2, 32.3)	1469	40.7 (38.9, 42.5)
Having a strong leader for America is more important than having a democracy.								
Do not agree	525	44.9 (41.7, 48.1)	387	59.9 (55.7, 64.0)	946	57.3 (54.5, 60.0)	2667	64.5 (62.7, 66.2)
Somewhat agree	253	24.1 (21.2, 27.0)	145	22.3 (18.9, 25.8)	382	25.8 (23.3, 28.2)	693	20.3 (18.8, 21.8)
Strongly or very strongly agree	337	31.0 (28.0, 34.1)	107	17.8 (14.5, 21.2)	241	17.0 (14.8, 19.2)	538	15.2 (13.9, 16.6)
Armed citizens should patrol polling places at election time.								
Do not agree	686	60.7 (57.5, 63.9)	545	82.6 (79.1, 86.1)	1384	86.9 (85.0, 88.9)	3624	90.7 (89.6, 91.9)
Somewhat agree	217	20.1 (17.5, 22.7)	71	12.5 (9.4, 15.5)	127	8.9 (7.3, 10.5)	172	5.7 (4.7, 6.6)
Strongly or very strongly agree	209	19.2 (16.6, 21.9)	24	5.0 (2.8, 7.1)	52	4.1 (2.9, 5.4)	108	3.6 (2.9, 4.3)

* Denotes self-identified Republicans who voted for Donald Trump in 2020 and agreed strongly or very strongly with the statement that “the 2020 election was stolen from Donald Trump, and Joe Biden is an illegitimate president.”

By wide margins, MAGA Republicans were more likely than others to agree strongly or very strongly that force or violence might be justified under three sets of circumstances: 1) to “protect American democracy…if elected leaders will not,” 2) to save “our American way of life” that is “disappearing so fast,” and 3) to “save our country” because “things have gotten so far off track” (Table 3, S2 Table in S1 File; p < 0.001 for all three comparisons). Among MAGA Republicans, strong or very strong support for these positions ranged from 26.3% to 46.0%. They were also much more likely to agree strongly or very strongly that “in the next few years, there will be civil war in the United States” (MAGA Republicans, 30.3%, 95% CI 27.2%, 33.4%; strong Republicans, 7.5%, 95% CI 5.1%, 9.9%; other Republicans, 10.8%, 95% CI 9.0%, 12.6%; non-Republicans, 11.2%, 95% CI 10.0%, 12.3%; p < 0.001) (Table 3, S2 Table in S1 File).

**Table 3:** Beliefs concerning the potential need for violence in the US, by study group.

Statement	Republicans						Non-Republicans	
	MAGA Republicans*		Strong Republicans		Other Republicans			
	Unweighted n	Weighted % (95% CI)	Unweighted n	Weighted % (95% CI)	Unweighted n	Weighted % (95% CI)	Unweighted n	Weighted % (95% CI)
If elected leaders will not protect American democracy, the people must do it themselves, even if it requires taking violent actions.								
Do not agree	301	27.2 (24.3, 30.1)	281	43.2 (39.0, 47.4)	804	49.3 (46.6, 52.1)	2441	58.7 (57.0, 60.5)
Somewhat agree	355	31.9 (28.9, 34.9)	232	36.6 (32.5, 40.6)	513	33.9 (31.3, 36.5)	984	27.5 (25.9, 29.1)
Strongly or very strongly agree	457	40.9 (37.6, 44.1)	122	20.3 (16.8, 23.7)	248	16.8 (14.7, 18.9)	467	13.8 (12.5, 15.1)
Our American way of life is disappearing so fast that we may have to use force to save it.								
Do not agree	195	16.9 (14.5, 19.4)	299	44.9 (40.7, 49.1)	881	54.5 (51.8, 57.3)	2891	71.3 (69.6, 72.9)
Somewhat agree	412	37.1 (33.9, 40.2)	232	37.0 (32.9, 41.1)	495	32.9 (30.3, 35.5)	716	19.9 (18.5, 21.4)
Strongly or very strongly agree	508	46.0 (42.7, 49.2)	105	18.1 (14.7, 21.5)	191	12.6 (10.7, 14.5)	292	8.8 (7.7, 9.9)
Because things have gotten so far off track, true American patriots may have to resort to violence in order to save our country.								
Do not agree	420	38.1 (34.9, 41.3)	442	67.7 (63.7, 71.7)	1211	75.2 (72.7, 77.7)	3418	85 (83.6, 86.4)
Somewhat agree	399	35.6 (32.5, 38.7)	160	25.4 (21.7, 29.2)	273	19.5 (17.2, 21.8)	373	11.1 (9.9, 12.4)
Strongly agree	296	26.3 (23.4, 29.2)	36	6.9 (4.5, 9.2)	77	5.3 (4.1, 6.5)	115	3.8 (3.1, 4.6)
In the next few years, there will be civil war in the United States.								
Do not agree	304	25.6 (22.8, 28.3)	343	53.1 (48.9, 57.3)	868	53.8 (51.1, 56.6)	2139	53.4 (51.6, 55.2)
Somewhat agree	495	44.1 (40.9, 47.4)	251	39.4 (35.3, 43.6)	538	35.4 (32.7, 38.0)	1368	35.4 (33.7, 37.1)
Strongly or very strongly agree	316	30.3 (27.2, 33.4)	42	7.5 (5.1, 9.9)	152	10.8 (9.0, 12.6)	380	11.2 (10.0, 12.3)

* Denotes self-identified Republicans who voted for Donald Trump in 2020 and agreed strongly or very strongly with the statement that “the 2020 election was stolen from Donald Trump, and Joe Biden is an illegitimate president.”

### Race and ethnicity and American society

Five items explored beliefs on race and “great replacement” thinking (Table 4, S3 Table in S1 File). MAGA Republicans were less likely than other Republicans and non-Republicans (but not strong Republicans) to agree strongly or very strongly with the statements that “white people benefit from advantages in society that Black people do not have” (MAGA Republicans, 6.5%, 95% CI 4.7%, 8.2%; strong Republicans, 7.0%, 95% CI 4.6%, 9.4%; other Republicans, 18.2%, 95% CI 16.0%, 20.4%; non-Republicans, 62.6%, 95% CI 60.9%, 64.3%; p < 0.001) and that “having more Black Americans, Latinos, and Asian Americans is good for the country” (MAGA Republicans, 20.1%, 95% CI 17.3%, 23.0%; strong Republicans, 21.6%, 95% CI 17.9%, 25.3%; other Republicans, 33.1%, 95% CI 30.4%, 35.7%; non-Republicans, 62.7%, 95% CI 61.0%, 64.4%; p < 0.001). They were far more likely than others to agree strongly or very strongly that “discrimination against whites is as big a problem as discrimination against Blacks and other minorities” (MAGA Republicans, 71.6%, 95% CI 68.7%, 74.5%; strong Republicans, 44.1%, 95% CI 39.9%, 48.3%; other Republicans, 33.3%, 95% CI 30.7%, 35.9%; non-Republicans, 10.6%, 95% CI 9.5%, 11.8%; p < 0.001) and that “in America, native-born white people are being replaced by immigrants” (MAGA Republicans, 51.0%, 95% CI 47.8%, 54.3%; strong Republicans, 23.1%, 95% CI 19.6%, 26.7%; other Republicans, 14.4%, 95% CI 12.5%, 16.3%; non-Republicans, 7.0%, 95% CI 6.0%, 8.0%; p < 0.001).

**Table 4:** Beliefs concerning race and ethnicity and American society, by study group.

Statement	Republicans						Non-Republicans	
	MAGA Republicans*		Strong Republicans		Other Republicans			
	Unweighted n	Weighted % (95% CI)	Unweighted n	Weighted % (95% CI)	Unweighted n	Weighted % (95% CI)	Unweighted n	Weighted % (95% CI)
White people benefit from advantages in society that Black people do not have.								
Do not agree	861	76.0 (73.2, 78.9)	422	66.6 (62.7, 70.6)	742	46.2 (43.5, 48.9)	437	11.1 (10.0, 12.2)
Somewhat agree	200	17.5 (15.0, 20.0)	180	26.4 (22.7, 30.0)	564	35.6 (33.0, 38.3)	1056	26.3 (24.7, 27.8)
Strongly or very strongly agree	58	6.5 (4.7, 8.2)	36	7.0 (4.6, 9.4)	262	18.2 (16.0, 20.4)	2410	62.6 (60.9, 64.3)
Straight white men hold far too much power in America.								
Do not agree	877	75.4 (72.4, 78.4)	522	80.4 (76.8, 83.9)	987	59.9 (57.1, 62.6)	786	19.5 (18.1, 20.9)
Somewhat agree	168	16.6 (14.0, 19.2)	85	13.6 (10.6, 16.6)	388	25.7 (23.2, 28.1)	1223	30.3 (28.7, 32.0)
Strongly or very strongly agree	73	8.0 (6.2, 9.9)	32	6.0 (3.7, 8.3)	189	14.5 (12.4, 16.5)	1889	50.1 (48.4, 51.9)
Discrimination against whites is as big a problem as discrimination against Blacks and other minorities.								
Do not agree	69	6.4 (4.8, 8.0)	114	18.1 (14.8, 21.3)	484	31.3 (28.7, 33.8)	2903	73.5 (71.9, 75.1)
Somewhat agree	247	22.0 (19.3, 24.6)	244	37.9 (33.8, 41.9)	565	35.4 (32.8, 38.1)	610	15.9 (14.6, 17.2)
Strongly or very strongly agree	806	71.6 (68.7, 74.5)	280	44.1 (39.9, 48.3)	518	33.3 (30.7, 35.9)	392	10.6 (9.5, 11.8)
Having more Black Americans, Latinos, and Asian Americans is good for the country.								
Do not agree	488	42.4 (39.2, 45.6)	218	32.3 (28.5, 36.2)	396	23.3 (21.1, 25.5)	362	9.3 (8.3, 10.4)
Somewhat agree	418	37.5 (34.3, 40.6)	294	46.1 (41.9, 50.3)	685	43.6 (40.9, 46.4)	1114	28.0 (26.4, 29.6)
Strongly or very strongly agree	196	20.1 (17.3, 23.0)	118	21.6 (17.9, 25.3)	472	33.1 (30.4, 35.7)	2406	62.7 (61.0, 64.4)
In America, native-born white people are being replaced by immigrants.								
Do not agree	180	17.1 (14.6, 19.6)	216	35.3 (31.2, 39.4)	738	49.6 (46.9, 52.4)	3028	76.5 (74.9, 78.0)
Somewhat agree	355	31.9 (28.9, 34.9)	274	41.6 (37.4, 45.7)	594	36.0 (33.4, 38.6)	635	16.5 (15.2, 17.9)
Strongly or very strongly agree	582	51.0 (47.8, 54.3)	149	23.1 (19.6, 26.7)	235	14.4 (12.5, 16.3)	238	7.0 (6.0, 8.0)

* Denotes self-identified Republicans who voted for Donald Trump in 2020 and agreed strongly or very strongly with the statement that “the 2020 election was stolen from Donald Trump, and Joe Biden is an illegitimate president.”

Three items addressed the central elements of the QAnon mythology and a separate religious belief (Table 5, S4 Table in S1 File). MAGA Republicans were much more likely than others to agree strongly or very strongly that US institutions “are controlled by a group of Satan-worshipping pedophiles who run a global child sex trafficking operation” (MAGA Republicans, 26.7%, 95% CI 23.7%, 29.7%; strong Republicans, 5.4%, 95% CI 3.3%, 7.5%; other Republicans, 6.5%, 95% CI 4.9%, 8.0%; non-Republicans, 5.6%, 95% CI 4.6%, 6.6%; p < 0.001) and that “a storm coming soon” will “sweep away the elites in power and restore the rightful leaders” (MAGA Republicans, 29.6%, 95% CI 26.5%, 32.6%; strong Republicans, 7.0%, 95% CI 4.6%, 9.4%; other Republicans, 5.7%, 95% CI 4.3%, 5.1%; non-Republicans, 6.3%, 95% CI 5.4%, 7.3%; p < 0.001). They were also more likely than others to agree strongly or very strongly that “the chaos in America today is evidence that we are living in what the Bible calls ‘the end times’” (MAGA Republicans, 37.8%, 95% CI 34.6%, 41.0%; strong Republicans, 21.0%, 95% CI 17.4%, 24.6%; other Republicans, 15.1%, 95% CI 13.0%, 17.2%; non-Republicans, 13.9%, 95% CI 12.6%, 15.2%; p < 0.001).

**Table 5:** Beliefs concerning QAnon and biblical “end times,” by study group.

Statement	Republicans						Non-Republicans	
	MAGA Republicans*		Strong Republicans		Other Republicans			
	Unweighted n	Weighted % (95% CI)	Unweighted n	Weighted % (95% CI)	Unweighted n	Weighted % (95% CI)	Unweighted n	Weighted % (95% CI)
The government, media, and financial worlds in the U.S. are controlled by a group of Satan-worshipping pedophiles who run a global child sex trafficking operation.								
Do not agree	550	46.7 (43.5, 50.0)	547	83.6 (80.2, 86.9)	1302	79.8 (77.4, 82.2)	3471	85.7 (84.3, 87.1)
Somewhat agree	285	26.6 (23.7, 29.5)	60	11.0 (8.1, 13.8)	182	13.7 (11.7, 15.7)	259	8.7 (7.5, 9.8)
Strongly or very strongly agree	269	26.7 (23.7, 29.7)	29	5.4 (3.3, 7.5)	80	6.5 (4.9, 8.0)	157	5.6 (4.6, 6.6)
There is a storm coming soon that will sweep away the elites in power and restore the rightful leaders.								
Do not agree	399	35.6 (32.4, 38.7)	449	70.1 (66.1, 74.0)	1195	74.6 (72.2, 77.1)	3175	78.9 (77.3, 80.4)
Somewhat agree	392	34.9 (31.8, 37.9)	147	22.9 (19.4, 26.5)	290	19.7 (17.4, 21.9)	504	14.8 (13.5, 16.1)
Strongly or very strongly agree	320	29.6 (26.5, 32.6)	38	7.0 (4.6, 9.4)	76	5.7 (4.3, 7.1)	192	6.3 (5.4, 7.3)
The chaos in America today is evidence that we are living in what the Bible calls “the end times.”								
Do not agree	327	28.3 (25.4, 31.2)	326	49.6 (45.3, 53.8)	884	54.6 (51.8, 57.3)	2730	67.1 (65.4, 68.8)
Somewhat agree	386	33.9 (30.8, 36.9)	192	29.4 (25.6, 33.3)	465	30.3 (27.8, 32.9)	698	19.0 (17.6, 20.5)
Strongly or very strongly agree	401	37.8 (34.6, 41.0)	121	21.0 (17.4, 24.6)	215	15.1 (13.0, 17.2)	470	13.9 (12.6, 15.2)

* Denotes self-identified Republicans who voted for Donald Trump in 2020 and agreed strongly or very strongly with the statement that “the 2020 election was stolen from Donald Trump, and Joe Biden is an illegitimate president.”

### Non-political violence

More than 90% of respondents in all groups saw violence as at least sometimes justified in self-defense or to prevent assaultive or self-inflicted injury to others. In adjusted models, MAGA Republicans were more likely than other Republicans and non-Republicans (but not strong Republicans) to view violence under those circumstances as usually or always justified (Table 6, S5 Table in S1 File; p < 0.001). They were more likely than all other groups to consider violence to prevent harm or damage to property as usually or always justified (MAGA Republicans, 54.5%, 95% CI 51.3%, 57.7%; strong Republicans, 43.6%, 95% CI 39.5%, 47.8%; other Republicans, 37.4%, 95% CI 34.7%, 40.1%; non-Republicans, 25.7%, 95% CI 24.2%, 27.3%; p < 0.001).

**Table 6:** Justification for violence in non-political situations, by study group.

In general, what do you think about the use of force or violence…	Republicans						Non-Republicans	
	MAGA Republicans*		Strong Republicans		Other Republicans			
	Unweighted n	Weighted % (95% CI)	Unweighted n	Weighted % (95% CI)	Unweighted n	Weighted % (95% CI)	Unweighted n	Weighted % (95% CI)
In self defense								
Never justified	9	1.0 (0.3, 1.7)	6	1.3 (0.2, 2.4)	15	0.9 (0.4, 1.4)	67	1.9 (1.4, 2.4)
Sometimes justified	96	8.9 (7.0, 10.8)	77	12.0 (9.3, 14.7)	245	16.2 (14.1, 18.3)	1041	26.6 (25.0, 28.2)
Usually or always justified	1022	90.1 (88.1, 92.1)	557	86.7 (83.8, 89.5)	1311	82.9 (80.8, 85.0)	2797	71.5 (69.9, 73.1)
To prevent someone from injuring or killing another person								
Never justified	8	0.7 (0.2, 1.2)	5	1.0 (0.1, 2.0)	16	1.1 (0.5, 1.6)	78	2.5 (1.9, 3.1)
Sometimes justified	94	9.4 (7.4, 11.4)	69	11.3 (8.6, 14.1)	226	15.7 (13.6, 17.8)	880	23.3 (21.8, 24.9)
Usually or always justified	1022	89.9 (87.9, 92.0)	565	87.6 (84.8, 90.5)	1323	83.2 (81.1, 85.4)	2935	74.2 (72.6, 75.8)
To prevent someone from injuring or killing themselves								
Never justified	53	5.1 (3.5, 6.6)	27	3.8 (2.2, 5.3)	63	3.9 (2.9, 4.9)	246	7.0 (6.0, 7.9)
Sometimes justified	296	26.1 (23.3, 28.9)	198	31.7 (27.8, 35.7)	516	33.8 (31.2, 36.5)	1400	36.9 (35.2, 38.6)
Usually or always justified	777	68.8 (65.8, 71.8)	413	64.5 (60.4, 68.5)	988	62.3 (59.6, 65.0)	2254	56.1 (54.3, 57.9)
To prevent harm or damage to property								
Never justified	81	7.5 (5.8, 9.2)	52	8.3 (5.9, 10.7)	160	10.0 (8.4, 11.6)	820	21.1 (19.6, 22.5)
Sometimes justified	427	38.0 (34.9, 41.1)	304	48.0 (43.8, 52.3)	822	52.6 (49.9, 55.3)	2083	53.2 (51.4, 54.9)
Usually or always justified	618	54.5 (51.3, 57.7)	284	43.6 (39.5, 47.8)	585	37.4 (34.7, 40.1)	992	25.7 (24.2, 27.3)
To win an argument								
Never justified	961	85.1 (82.9, 87.4)	578	89.5 (86.8, 92.2)	1420	90.2 (88.5, 91.8)	3505	88.2 (87.0, 89.4)
Sometimes justified	130	11.6 (9.5, 13.6)	50	7.9 (5.6, 10.1)	121	7.8 (6.3, 9.3)	278	8.3 (7.2, 9.4)
Usually or always justified	34	3.3 (2.1, 4.5)	12	2.6 (1.0, 4.3)	27	2.0 (1.2, 2.9)	119	3.5 (2.8, 4.2)
In response to an insult								
Never justified	953	83.8 (81.3, 86.2)	573	88.1 (85.2, 91.1)	1374	86.4 (84.4, 88.3)	3348	83.2 (81.8, 84.7)
Sometimes justified	127	12.0 (9.8, 14.1)	48	8.6 (6.1, 11.2)	158	11.0 (9.2, 12.8)	414	12.5 (11.3, 13.8)
Usually or always justified	46	4.3 (3.0, 5.6)	17	3.2 (1.6, 4.8)	37	2.6 (1.7, 3.5)	138	4.2 (3.5, 5.0)
To get respect								
Never justified	995	87.8 (85.7, 90.0)	598	92.3 (89.8, 94.8)	1447	91.1 (89.4, 92.7)	3506	88.2 (86.9, 89.4)
Sometimes justified	92	8.2 (6.4, 10.0)	27	5.2 (3.1, 7.2)	91	6.5 (5.1, 7.9)	254	7.7 (6.7, 8.7)
Usually or always justified	40	4.0 (2.7, 5.3)	14	2.6 (1.1, 4.0)	30	2.4 (1.5, 3.3)	143	4.2 (3.4, 4.9)

* Denotes self-identified Republicans who voted for Donald Trump in 2020 and agreed strongly or very strongly with the statement that “the 2020 election was stolen from Donald Trump, and Joe Biden is an illegitimate president.”

Conversely, more than 80% of respondents across all groups reported that violence to win an argument, respond to an insult, or get respect was never justified, and fewer than 5% of respondents in any group saw viewed such violence as usually or always justified. In fully adjusted models, there were no significant differences between groups (Table 6, S5 Table in S1 File).

### Political violence

#### In general and to advance specific objectives

Support for political violence as usually or always justified “in general” was uncommon in all groups, but slightly less uncommon among MAGA Republicans than among other Republicans and non-Republicans (MAGA Republicans, 3.3%, 95% CI 2.0%, 4.6%; strong Republicans, 2.5%, 95% CI 0.6%, 4.3%; other Republicans, 1.1%, 95% CI 0.5%, 1.8%; non-Republicans, 2.5%, 95% CI 1.9%, 3.2%) (Table 7, S6 Table in S1 File; p = 0.006).

**Table 7:** Justification for political violence “in general” and to advance nine specific political objectives, by study group.

What do you think about the use of force or violence in the following situations?	Republicans						Non-Republicans	
	MAGA Republicans*		Strong Republicans		Other Republicans			
	Unweighted n	Weighted % (95% CI)	Unweighted n	Weighted % (95% CI)	Unweighted n	Weighted % (95% CI)	Unweighted n	Weighted % (95% CI)
In general…to advance an important political objective that you support								
Never justified	873	77.6 (74.9, 80.4)	547	83.6 (80.2, 87.0)	1350	83.9 (81.7, 86.0)	3233	79.2 (77.6, 80.7)
Sometimes justified	219	19.1 (16.5, 21.6)	85	13.9 (10.9, 16.9)	207	15.0 (12.9, 17.1)	607	18.3 (16.9, 19.8)
Usually or always justified	33	3.3 (2.0, 4.6)	8	2.5 (0.6, 4.3)	14	1.1 (0.5, 1.8)	72	2.5 (1.9, 3.2)
To advance at least 1 of 17 specific political objectives								
Never justified	100	9.4 (7.5, 11.3)	82	12.3 (9.7, 15.0)	271	17.5 (15.5, 19.6)	1056	26.1 (24.6, 27.6)
Sometimes justified	360	32.4 (29.3, 35.4)	312	49.3 (45.1, 53.6)	807	51.0 (48.3, 53.7)	1939	48.8 (47.0, 50.5)
Usually or always justified	668	58.2 (55.0, 61.4)	246	38.3 (34.2, 42.4)	493	31.5 (28.9, 34.0)	921	25.1 (23.6, 26.7)
To return Donald Trump to the presidency this year								
Never justified	805	71.5 (68.5, 74.5)	572	89.3 (86.6, 91.9)	1462	92.6 (91.1, 94.1)	3687	93.4 (92.4, 94.4)
Sometimes justified	144	12.9 (10.7, 15.0)	35	5.3 (3.4, 7.1)	61	4.2 (3.1, 5.3)	109	3.6 (2.8, 4.4)
Usually or always justified	166	15.6 (13.1, 18.1)	32	5.4 (3.4, 7.4)	41	3.2 (2.2, 4.3)	93	3.0 (2.3, 3.7)
To stop an election from being stolen								
Never justified	549	49.8 (46.6, 53.1)	462	72.3 (68.6, 76.1)	1214	76.8 (74.4, 79.2)	3242	82.3 (81.0, 83.7)
Sometimes justified	319	27.8 (24.9, 30.7)	126	19.7 (16.4, 23.1)	277	18.4 (16.2, 20.6)	453	11.9 (10.7, 13.0)
Usually or always justified	249	22.4 (19.6, 25.2)	51	7.9 (5.6, 10.3)	72	4.8 (3.6, 6.0)	201	5.8 (4.9, 6.7)
To stop people who do not share my beliefs from voting								
Never justified	1050	92.9 (91.2, 94.6)	614	95.4 (93.4, 97.5)	1509	95.7 (94.5, 96.9)	3708	93.7 (92.8, 94.7)
Sometimes justified	55	5.5 (4.0, 7.1)	16	2.9 (1.3, 4.4)	47	3.6 (2.5, 4.7)	113	3.6 (2.8, 4.3)
Usually or always justified	16	1.5 (0.7, 2.4)	8	1.7 (0.3, 3.0)	10	0.8 (0.3, 1.3)	79	2.7 (2.0, 3.4)
To prevent discrimination based on race or ethnicity								
Never justified	762	68.2 (65.1, 71.2)	466	71.8 (67.9, 75.7)	1084	67.6 (65.0, 70.2)	2466	60.6 (58.8, 62.4)
Sometimes justified	255	22.7 (20.0, 25.5)	136	21.9 (18.4, 25.5)	398	26.6 (24.1, 29.1)	1096	29.4 (27.8, 31.1)
Usually or always justified	101	9.1 (7.2, 10.9)	36	6.2 (4.0, 8.5)	83	5.8 (4.5, 7.2)	331	10.0 (8.8, 11.1)
To preserve an American way of life based on Western European traditions								
Never justified	589	54.0 (50.7, 57.2)	418	66.2 (62.2, 70.2)	1117	72.9 (70.5, 75.3)	3286	84.2 (82.9, 85.5)
Sometimes justified	384	32.9 (29.9, 35.9)	174	26.7 (23.0, 30.4)	377	23.3 (21.0, 25.6)	474	12.0 (10.9, 13.1)
Usually or always justified	139	13.1 (10.8, 15.4)	43	7.1 (4.7, 9.4)	63	3.8 (2.8, 4.9)	124	3.8 (3.0, 4.5)
To preserve the American way of life l believe in								
Never justified	324	30.2 (27.2, 33.2)	268	42.7 (38.5, 46.9)	777	51.0 (48.2, 53.7)	2586	65.8 (64.1, 67.5)
Sometimes justified	472	42.0 (38.8, 45.2)	263	40.1 (36.0, 44.2)	626	39.2 (36.5, 41.9)	1036	26.4 (24.9, 28.0)
Usually or always justified	325	27.7 (24.8, 30.6)	108	17.2 (13.9, 20.5)	165	9.9 (8.3, 11.4)	284	7.7 (6.8, 8.7)
To oppose Americans who do not share my beliefs								
Never justified	1000	88.8 (86.8, 90.9)	599	93.1 (90.9, 95.3)	1458	92.0 (90.5, 93.5)	3571	89.5 (88.3, 90.7)
Sometimes justified	105	9.5 (7.6, 11.3)	33	5.2 (3.3, 7.0)	96	6.9 (5.5, 8.4)	254	7.7 (6.7, 8.7)
Usually or always justified	19	1.7 (0.9, 2.6)	8	1.8 (0.4, 3.1)	16	1.1 (0.5, 1.6)	82	2.8 (2.1, 3.6)
To oppose the government when it does not share my beliefs								
Never justified	814	73.5 (70.6, 76.4)	523	81.2 (77.8, 84.6)	1300	81.4 (79.1, 83.7)	3376	83.9 (82.5, 85.3)
Sometimes justified	254	22.0 (19.3, 24.7)	97	15.7 (12.6, 18.9)	239	16.5 (14.4, 18.7)	430	13.1 (11.8, 14.3)
Usually or always justified	45	4.5 (3.1, 5.9)	18	3.1 (1.5, 4.6)	27	2.1 (1.2, 3.0)	92	3.0 (2.3, 3.7)
To oppose the government when it tries to take private land for public purposes								
Never justified	441	39.8 (36.7, 43.0)	348	53.2 (49.0, 57.4)	949	60.0 (57.3, 62.8)	2806	69.7 (68.0, 71.4)
Sometimes justified	448	38.4 (35.3, 41.5)	241	37.7 (33.6, 41.8)	490	30.8 (28.3, 33.4)	876	23.8 (22.2, 25.3)
Usually or always justified	226	21.8 (19.0, 24.6)	49	9.1 (6.4, 11.8)	121	9.1 (7.4, 10.9)	209	6.5 (5.6, 7.5)

* Denotes self-identified Republicans who voted for Donald Trump in 2020 and agreed strongly or very strongly with the statement that “the 2020 election was stolen from Donald Trump, and Joe Biden is an illegitimate president.”

Regardless of their response on the justification for political violence in general, respondents were asked about the justification for political violence to advance specific political objectives (Tables 7 and 8, S6 and S7 Tables in S1 File). Nearly a third of respondents (32.6%, 95% CI 31.3%, 33.8%) considered violence to be usually or always justified for at least one of these 17 specific political objectives, and MAGA Republicans were more likely than others to do so (MAGA Republicans, 58.2%, 95% CI 55.0%, 61.4%; strong Republicans, 38.3%, 95% CI 34.2%, 42.4%; other Republicans, 31.5%, 95% CI 28.9%, 34.0%; non-Republicans, 25.1%, 95% CI 23.6%, 26.7%; p < 0.001).

**Table 8:** Justification for political violence to advance 8 additional specific objectives, by study group (these objectives were paired, with respondents randomized 1:1 to see 1 item in each pair).

What do you think about the use of force or violence in the following situations?	Republicans						Non-Republicans	
	MAGA Republicans*		Strong Republicans		Other Republicans			
	Unweighted n	Weighted % (95% CI)	Unweighted n	Weighted % (95% CI)	Unweighted n	Weighted % (95% CI)	Unweighted n	Weighted % (95% CI)
To stop voter fraud								
Never justified	290	52.4 (47.9, 57.0)	241	76.7 (71.9, 81.6)	604	75.6 (72.2, 79.0)	1605	82.0 (79.9, 84.0)
Sometimes justified	139	24.4 (20.5, 28.3)	50	15.4 (11.2, 19.5)	147	19.4 (16.2, 22.6)	196	11.0 (9.4, 12.7)
Usually or always justified	134	23.1 (19.2, 27.1)	29	7.9 (4.9, 10.8)	47	5.0 (3.5, 6.6)	124	7.0 (5.6, 8.4)
To stop voter intimidation								
Never justified	281	52.1 (47.4, 56.7)	186	58.3 (52.3, 64.2)	506	65.3 (61.6, 69.0)	1223	61.9 (59.5, 64.3)
Sometimes justified	179	30.6 (26.4, 34.7)	82	26.2 (20.9, 31.5)	216	28.6 (25.0, 32.1)	576	29.2 (26.9, 31.5)
Usually or always justified	100	17.4 (13.8, 21.0)	52	15.6 (11.2, 19.9)	50	6.2 (4.3, 8.0)	184	8.9 (7.5, 10.3)
To reinforce the police								
Never justified	96	19.5 (15.7, 23.3)	68	22.3 (17.3, 27.4)	250	34.2 (30.5, 38.0)	1034	54.6 (52.1, 57.1)
Sometimes justified	209	36.9 (32.4, 41.4)	148	45.8 (39.9, 51.7)	380	47.6 (43.8, 51.5)	708	35.8 (33.4, 38.2)
Usually or always justified	253	43.5 (38.9, 48.2)	104	31.9 (26.4, 37.4)	153	18.1 (15.2, 21.0)	190	9.7 (8.2, 11.1)
To stop police violence								
Never justified	285	50.7 (46.2, 55.2)	183	55.4 (49.4, 61.4)	411	50.3 (46.4, 54.2)	873	41.4 (38.9, 43.8)
Sometimes justified	201	36.1 (31.8, 40.5)	115	37.6 (31.7, 43.5)	300	39.7 (35.9, 43.5)	867	45.1 (42.6, 47.6)
Usually or always justified	79	13.2 (10.3, 16.0)	22	6.9 (3.9, 10.0)	76	10.0 (7.7, 12.3)	236	13.6 (11.8, 15.4)
Stop illegal immigration								
Never justified	140	26.0 (22.0, 30.0)	129	42.3 (36.4, 48.2)	417	54.3 (50.4, 58.1)	1524	77.2 (75.0, 79.3)
Sometimes justified	214	39.5 (35.0, 44.0)	129	40.4 (34.5, 46.3)	299	36.4 (32.7, 40.1)	316	16.8 (14.9, 18.7)
Usually or always justified	202	34.5 (30.1, 38.9)	54	17.3 (12.8, 21.8)	84	9.3 (7.2, 11.5)	102	6.1 (4.8, 7.3)
To keep borders open								
Never justified	402	70.4 (66.1, 74.7)	223	69.7 (64.2, 75.1)	525	68.7 (65.1, 72.3)	1324	66.0 (63.6, 68.5)
Sometimes justified	95	17.2 (13.7, 20.6)	71	19.5 (15.1, 24.0)	187	23.9 (20.6, 27.2)	534	27.9 (25.6, 30.2)
Usually or always justified	67	12.5 (9.2, 15.8)	32	10.8 (6.8, 14.8)	56	7.4 (5.4, 9.4)	108	6.1 (4.8, 7.3)
To stop a protest								
Never justified	214	38.6 (34.2, 43.1)	142	46.1 (40.2, 52.0)	379	51.5 (47.6, 55.5)	1323	67.9 (65.5, 70.3)
Sometimes justified	272	49.6 (45.0, 54.2)	155	45.8 (40.0, 51.7)	335	43.5 (39.5, 47.4)	543	28.2 (25.9, 30.4)
Usually or always justified	72	11.7 (8.8, 14.7)	27	8.1 (5.0, 11.3)	36	5.0 (3.3, 6.7)	68	3.9 (2.8, 5.0)
To support a protest								
Never justified	466	81.4 (77.9, 85.0)	278	87.2 (83.0, 91.4)	685	83.2 (80.4, 86.1)	1558	76.3 (74.1, 78.5)
Sometimes justified	77	14.2 (11.0, 17.4)	27	8.8 (5.3, 12.3)	115	14.4 (11.8, 17.1)	336	19.0 (17.0, 21.1)
Usually or always justified	23	4.3 (2.5, 6.2)	11	4.0 (1.5, 6.6)	19	2.3 (1.2, 3.4)	79	4.7 (3.6, 5.8)

* Denotes self-identified Republicans who voted for Donald Trump in 2020 and agreed strongly or very strongly with the statement that “the 2020 election was stolen from Donald Trump, and Joe Biden is an illegitimate president.”

For eight of these 17 political objectives considered individually, MAGA Republicans were more likely than each of the other groups to believe that violence was usually or always justified (Tables 7 and 8, S6 and S7 Tables in S1 File). The prevalence of usually/always justification among MAGA Republicans for these eight objectives ranged from 13.1% (95% CI 10.8%, 15.4%) for “to preserve an American way of life based on Western European traditions” to 43.5% (95% CI 38.9%, 48.2) for “to reinforce the police.”

MAGA Republicans also endorsed violence for a greater number of specific political objectives than others did. The mean number of objectives for which violence was seen as usually or always justified was 2.0 (95% CI 1.8, 2.1) for MAGA Republicans, 1.1 (95% CI 0.9, 1.3) for strong Republicans, 0.7 (95% CI 0.6, 0.8) for other Republicans, and 0.7 (95% CI 0.7, 0.8) for non-Republicans (p < 0.001).

#### Personal willingness to engage in political violence

Among all respondents, only small proportions in each group (<4%) reported that they would be very or completely willing to damage property or threaten, injure, or kill a person, and MAGA Republicans were not more likely than others to report such willingness (Table 9, S8 Table in S1 File).

**Table 9:** Personal willingness to engage in political violence, by type of violence and study group.

In a situation where you think force or violence is justified to advance an important political objective…How willing would you personally be to use force or violence in each of these ways?	Republicans						Non-Republicans	
	MAGA Republicans*		Strong Republicans		Other Republicans			
	Unweighted n	Weighted % (95% CI)	Unweighted n	Weighted % (95% CI)	Unweighted n	Weighted % (95% CI)	Unweighted n	Weighted % (95% CI)
To damage property								
Not asked the question	100	9.5 (7.6, 11.4)	82	12.3 (9.7, 15.0)	271	17.5 (15.5, 19.6)	1056	26.2 (24.7, 27.7)
Not willing	906	80.1 (77.5, 82.8)	514	79.2 (75.7, 82.7)	1194	75.1 (72.8, 77.5)	2435	61.6 (59.8, 63.3)
Somewhat willing	84	7.9 (6.0, 9.7)	31	5.6 (3.4, 7.7)	82	5.5 (4.3, 6.8)	307	8.7 (7.7, 9.8)
Very or completely willing	27	2.5 (1.5, 3.5)	13	2.9 (1.1, 4.6)	23	1.8 (1.0, 2.6)	107	3.5 (2.8, 4.3)
To threaten or intimidate a person								
Not asked the question	100	9.5 (7.6, 11.5)	82	12.4 (9.7, 15.0)	271	17.5 (15.5, 19.6)	1056	26.2 (24.7, 27.7)
Not willing	899	79.3 (76.6, 82.0)	515	79.8 (76.4, 83.3)	1179	73.8 (71.4, 76.3)	2556	64.6 (62.9, 66.3)
Somewhat willing	95	8.7 (6.8, 10.6)	29	5.3 (3.2, 7.4)	100	7.3 (5.7, 8.9)	233	7.1 (6.1, 8.1)
Very or completely willing	23	2.6 (1.4, 3.7)	12	2.5 (1.0, 4.1)	19	1.3 (0.7, 2.0)	60	2.1 (1.5, 2.7)
To injure a person								
Not asked the question	100	9.5 (7.6, 11.5)	82	12.4 (9.7, 15.0)	271	17.6 (15.5, 19.6)	1056	26.2 (24.7, 27.7)
Not willing	911	81.2 (78.6, 83.7)	516	79.6 (76.1, 83.1)	1196	75.1 (72.6, 77.5)	2608	66.2 (64.5, 67.9)
Somewhat willing	82	7.1 (5.5, 8.8)	32	6.2 (4.0, 8.5)	79	5.7 (4.3, 7.1)	172	5.4 (4.5, 6.3)
Very or completely willing	23	2.2 (1.2, 3.1)	9	1.8 (0.5, 3.1)	21	1.7 (0.8, 2.5)	65	2.2 (1.6, 2.8)
To kill a person								
Not asked the question	100	9.5 (7.6, 11.4)	82	12.4 (9.7, 15.0)	271	17.6 (15.5, 19.6)	1056	26.2 (24.7, 27.8)
Not willing	936	83.4 (81.0, 85.8)	533	82.8 (79.6, 86.0)	1229	77.6 (75.3, 79.9)	2684	68.4 (66.8, 70.1)
Somewhat willing	55	4.6 (3.3, 5.9)	15	2.9 (1.4, 4.4)	39	2.9 (1.9, 3.8)	95	3.1 (2.4, 3.8)
Very or completely willing	28	2.5 (1.5, 3.5)	9	2.0 (0.5, 3.4)	29	2.0 (1.2, 2.8)	68	2.2 (1.6, 2.8)

* Denotes self-identified Republicans who voted for Donald Trump in 2020 and agreed strongly or very strongly with the statement that “the 2020 election was stolen from Donald Trump, and Joe Biden is an illegitimate president.”

Table 10 and S9 Table in S1 File present findings for violence against others because of occupational or social characteristics. Among all respondents, no more than 3% in any group reported that they were very or completely willing to use force or violence against the specified groups, and MAGA Republicans were not more willing than others (Table 10, S9 Table in S1 File).

**Table 10:** Personal willingness to engage in political violence, by target of violence and study group.

In a situation where you think force or violence is justified to advance an important political objective…How willing would you personally be to use force or violence against a person because they are…	Republicans						Non-Republicans	
	MAGA Republicans*		Strong Republicans		Other Republicans			
	Unweighted n	Weighted % (95% CI)	Unweighted n	Weighted % (95% CI)	Unweighted n	Weighted % (95% CI)	Unweighted n	Weighted % (95% CI)
An elected federal or state government official								
Not asked the question	100	9.6 (7.6, 11.5)	82	12.4 (9.7, 15.0)	271	17.6 (15.6, 19.7)	1056	26.3 (24.7, 27.8)
Not willing	905	80.3 (77.6, 83.0)	523	82.1 (79.0, 85.3)	1222	77.5 (75.2, 79.8)	2644	67.3 (65.7, 69.0)
Somewhat willing	81	7.4 (5.6, 9.2)	20	3.1 (1.7, 4.5)	59	4.0 (2.9, 5.1)	141	4.5 (3.7, 5.3)
Very or completely willing	26	2.7 (1.5, 4.0)	12	2.4 (0.9, 3.9)	10	0.9 (0.2, 1.5)	55	1.9 (1.3, 2.5)
An elected local government official								
Not asked the question	100	9.6 (7.6, 11.5)	82	12.4 (9.7, 15.0)	271	17.7 (15.6, 19.8)	1056	26.3 (24.8, 27.9)
Not willing	918	82.5 (80.0, 85.0)	527	82.8 (79.6, 85.9)	1222	77.3 (75.0, 79.6)	2655	67.8 (66.1, 69.5)
Somewhat willing	69	6.1 (4.6, 7.6)	19	3.3 (1.7, 5.0)	54	4.0 (2.8, 5.2)	125	4.1 (3.3, 4.8)
Very or completely willing	19	1.8 (0.9, 2.7)	9	1.5 (0.4, 2.6)	12	1.0 (0.4, 1.7)	54	1.8 (1.3, 2.4)
An election worker, such as a poll worker or vote counter								
Not asked the question	100	9.5 (7.6, 11.5)	82	12.4 (9.7, 15.0)	271	17.6 (15.5, 19.7)	1056	26.3 (24.7, 27.8)
Not willing	963	85.7 (83.4, 88.1)	534	83.6 (80.5, 86.7)	1260	79.8 (77.6, 82.0)	2717	69.7 (68.0, 71.3)
Somewhat willing	35	3.3 (2.0, 4.6)	16	2.5 (1.2, 3.7)	27	2.0 (1.2, 2.9)	73	2.3 (1.7, 2.9)
Very or completely willing	15	1.5 (0.7, 2.3)	7	1.6 (0.2, 2.9)	7	0.5 (0.1, 0.9)	47	1.7 (1.2, 2.3)
A public health official								
Not asked the question	100	9.5 (7.6, 11.5)	82	12.4 (9.8, 15.1)	271	17.6 (15.5, 19.7)	1056	26.3 (24.8, 27.9)
Not willing	938	83.6 (81.2, 86.0)	526	82.6 (79.4, 85.8)	1235	78.4 (76.1, 80.6)	2713	69.7 (68.1, 71.3)
Somewhat willing	59	5.2 (3.8, 6.6)	18	3.5 (1.7, 5.3)	48	3.5 (2.5, 4.6)	71	2.3 (1.7, 2.9)
Very or completely willing	16	1.7 (0.8, 2.5)	8	1.5 (0.4, 2.6)	8	0.5 (0.1, 0.9)	50	1.7 (1.2, 2.3)
A member of the military or National Guard								
Not asked the question	100	9.6 (7.6, 11.5)	82	12.4 (9.7, 15.0)	271	17.6 (15.5, 19.7)	1056	26.3 (24.7, 27.8)
Not willing	943	84.0 (81.5, 86.4)	534	84.0 (81.0, 87.0)	1236	78.5 (76.3, 80.8)	2634	67.2 (65.5, 68.8)
Somewhat willing	50	4.6 (3.3, 6.0)	13	2.2 (1.0, 3.5)	41	2.9 (1.9, 3.8)	151	4.6 (3.8, 5.4)
Very or completely willing	18	1.8 (0.8, 2.9)	8	1.4 (0.3, 2.4)	13	1.0 (0.4, 1.6)	54	2.0 (1.4, 2.6)
A police officer								
Not asked the question	100	9.5 (7.6, 11.5)	82	12.4 (9.7, 15.0)	271	17.7 (15.6, 19.8)	1056	26.3 (24.7, 27.8)
Not willing	957	85.2 (82.8, 87.6)	537	83.7 (80.6, 86.8)	1222	77.9 (75.6, 80.1)	2581	65.7 (64.0, 67.4)
Somewhat willing	35	3.2 (2.1, 4.3)	10	1.7 (0.6, 2.9)	51	3.5 (2.5, 4.5)	181	5.4 (4.5, 6.2)
Very or completely willing	21	2.1 (1.0, 3.2)	10	2.2 (0.7, 3.7)	13	0.9 (0.4, 1.5)	77	2.7 (2.0, 3.4)
A person who does not share your race or ethnicity								
Not asked the question	100	9.6 (7.6, 11.5)	82	12.4 (9.7, 15.0)	271	17.6 (15.6, 19.7)	1056	26.3 (24.8, 27.8)
Not willing	966	86.5 (84.3, 88.7)	538	84.0 (80.9, 87.1)	1251	79.6 (77.4, 81.8)	2713	69.3 (67.6, 70.9)
Somewhat willing	32	2.7 (1.7, 3.7)	10	2.0 (0.6, 3.4)	29	2.0 (1.2, 2.7)	77	2.7 (2.0, 3.4)
Very or completely willing	13	1.3 (0.5, 2.0)	9	1.6 (0.5, 2.7)	10	0.8 (0.3, 1.4)	46	1.7 (1.2, 2.3)
A person who does not share your religion								
Not asked the question	100	9.5 (7.6, 11.5)	82	12.4 (9.7, 15.1)	271	17.6 (15.5, 19.7)	1056	26.3 (24.8, 27.8)
Not willing	980	87.1 (84.8, 89.4)	538	84.6 (81.5, 87.6)	1257	79.7 (77.5, 81.9)	2713	69.5 (67.8, 71.1)
Somewhat willing	25	2.6 (1.4, 3.9)	8	1.4 (0.4, 2.5)	26	2.0 (1.2, 2.9)	64	2.2 (1.6, 2.8)
Very or completely willing	8	0.7 (0.2, 1.2)	9	1.6 (0.4, 2.9)	8	0.6 (0.2, 1.1)	57	2.1 (1.5, 2.7)
A person who does not share your political beliefs								
Not asked the question	100	9.6 (7.6, 11.5)	82	12.4 (9.7, 15.0)	271	17.6 (15.5, 19.7)	1056	26.3 (24.7, 27.8)
Not willing	954	85.5 (83.3, 87.8)	540	84.5 (81.5, 87.5)	1249	79.1 (76.9, 81.4)	2670	68.2 (66.5, 69.8)
Somewhat willing	44	3.7 (2.5, 4.8)	6	1.0 (0.2, 1.9)	39	2.8 (1.9, 3.7)	126	4.0 (3.3, 4.8)
Very or completely willing	13	1.2 (0.5, 1.9)	10	2.1 (0.6, 3.6)	6	0.5 (0.1, 0.9)	44	1.5 (1.0, 2.0)

* Denotes self-identified Republicans who voted for Donald Trump in 2020 and agreed strongly or very strongly with the statement that “the 2020 election was stolen from Donald Trump, and Joe Biden is an illegitimate president.”

#### Possession and use of firearms

Finally, all 7,255 respondents were asked, regardless of their position on political violence or firearm ownership status, to predict the likelihood of their future use of a firearm “in a situation where you think force or violence is justified to advance an important political objective” (Table 11, S10 Table in S1 File). MAGA Republicans were more likely than others to report that it was very or extremely likely in such a situation that they would be “armed with a gun” (MAGA Republicans, 18.6%, 95% CI 15.9%, 21.2%; strong Republicans, 9.5%, 95% CI 6.6%, 12.4%; other Republicans, 8.1%, 95% CI 6.5%, 9.7%; non-Republicans, 4.7%, 95% CI 3.9%, 5.5%; p < 0.001) and would “carry a gun openly” (MAGA Republicans, 9.3%, 95% CI 7.1%, 11.5%; strong Republicans, 4.0%, 95% CI 2.1%, 5.9%; other Republicans, 3.2%, 95% CI 2.2%, 4.3%; non-Republicans, 2.6%, 95% CI 1.9%, 3.2%; p < 0.001). Few respondents thought it very or extremely likely that they would “threaten someone with a gun” or “shoot someone with a gun,” and there were no significant differences between groups.

**Table 11:** Future likelihood of firearm possession and use in a situation where political violence is perceived as justified, by study group.

Thinking now about the future and all the changes it might bring, how likely is it that you will use a gun in any of the following ways in the next few years—in a situation where you think force or violence is justified to advance an important political objective?	Republicans						Non-Republicans	
	MAGA Republicans*		Strong Republicans		Other Republicans			
	Unweighted n	Weighted % (95% CI)	Unweighted n	Weighted % (95% CI)	Unweighted n	Weighted % (95% CI)	Unweighted n	Weighted % (95% CI)
I will be armed with a gun.								
Not likely	761	65.8 (62.6, 69.0)	517	78.6 (75.0, 82.3)	1275	79.2 (76.8, 81.6)	3479	87.2 (85.9, 88.4)
Somewhat likely	157	15.6 (13.2, 18.1)	75	11.9 (9.1, 14.6)	181	12.7 (10.7, 14.6)	275	8.1 (7.1, 9.1)
Very or extremely likely	191	18.6 (15.9, 21.2)	44	9.5 (6.6, 12.4)	107	8.1 (6.5, 9.7)	148	4.7 (3.9, 5.5)
I will carry a gun openly, so that people know I am armed.								
Not likely	921	80.4 (77.6, 83.3)	571	88.8 (86.0, 91.7)	1428	90.5 (88.8, 92.2)	3687	93.4 (92.5, 94.4)
Somewhat likely	101	10.3 (8.2, 12.4)	44	7.2 (4.9, 9.4)	90	6.3 (4.9, 7.7)	135	4.0 (3.3, 4.7)
Very or extremely likely	86	9.3 (7.1, 11.5)	20	4.0 (2.1, 5.9)	44	3.2 (2.2, 4.3)	77	2.6 (1.9, 3.2)
I will threaten someone with a gun.								
Not likely	1092	98.0 (97.0, 99.0)	624	97.7 (96.1, 99.3)	1545	98.7 (98.0, 99.3)	3828	97.7 (97.2, 98.3)
Somewhat likely	14	1.2 (0.5, 1.9)	5	0.9 (0.0, 1.9)	15	1.1 (0.5, 1.7)	37	1.2 (0.8, 1.7)
Very or extremely likely	6	0.8 (0.1, 1.4)	5	1.3 (0.0, 2.6)	2	0.2 (0.0, 0.5)	31	1.0 (0.6, 1.4)
I will shoot someone with a gun.								
Not likely	1064	95.5 (94.1, 96.9)	616	95.9 (93.9, 97.9)	1525	97.4 (96.5, 98.2)	3789	96.3 (95.6, 97.1)
Somewhat likely	41	3.8 (2.5, 5.1)	14	2.9 (1.2, 4.6)	28	2.0 (1.2, 2.8)	74	2.6 (1.9, 3.2)
Very or extremely likely	6	0.7 (0.1, 1.4)	6	1.2 (0.1, 2.3)	10	0.7 (0.2, 1.1)	35	1.1 (0.7, 1.5)

* Denotes self-identified Republicans who voted for Donald Trump in 2020 and agreed strongly or very strongly with the statement that “the 2020 election was stolen from Donald Trump, and Joe Biden is an illegitimate president.”

### Sensitivity analysis

Results excluding the 447 respondents who failed the attentiveness check differed only slightly from those presented here and are available on request.

## Discussion

This study applies a public health approach to political violence. It uses standard methods to investigate variation in self-reported support for and willingness to engage in political violence, which are plausible proximate markers of risk for committing political violence [33]. It also assesses variation in prevalence of extreme beliefs—including the QAnon delusion and great replacement thinking, that have been linked to political violence in specific cases [34–36] and can be considered as potential indicators of risk for political violence.

MAGA Republicans, the group of primary interest, were defined here as self-identified Republicans who voted for Donald Trump in 2020 and strongly or very strongly denied the results of that election [10–12]. We estimate that, using this definition, MAGA Republicans account for approximately one-third (33.6%) of Republicans and 15.0% of the population. A different definition would produce a different estimate. For example, in a poll conducted in late November 2022 [37], 40% of Republicans answered yes to the question, “Do you identify as a MAGA Republican?”

This study first considered the question, Are MAGA Republicans a distinct subset of Republican party affiliates, other than on the characteristics incorporated in their definition? The question arose from critical reactions to the definition of MAGA Republican used by President Biden and adapted here; those reactions implied that, in essence, MAGA Republicans could not be distinguished from other Republicans [13, 17–20]. The study’s findings suggest that MAGA Republicans, as defined, are a distinct minority—more likely than other Republicans to endorse racist and delusional beliefs, sometimes by very wide margins.

The second question this study considered was, Are MAGA Republicans more likely than others to see political violence as justified and to be willing to engage personally in political violence? The analysis reveals that MAGA Republicans are substantially more likely than others to believe that general social and cultural conditions may create a need for violence and to expect civil war in the US in the next few years. They are more likely to consider violence as usually or always justified to advance at least 1 of 17 specific political objectives and to endorse violence to advance 8 of those 17 objectives considered individually. They are not, however, more likely than members of other groups to report that they are very or completely willing to engage in political violence. And while MAGA Republicans are at least twice as likely as members of other groups to consider it very or extremely likely that they will be armed and carry firearms openly in a future setting in which they consider political violence to be justified, they are not more likely than others to predict that they will use a firearm to threaten or shoot someone in such a situation.

The findings of this analysis are consistent with those of prior academic studies, not focused on a specified political group, that have found associations between support for Donald Trump and racist beliefs [38, 39] and, in 1 instance [39], political violence.

Based on this study’s findings, assessments by law enforcement experts in violent domestic extremism [7, 33, 34], and prior research [1–5, 39], concern about the potential for political violence among MAGA Republicans, as defined here, appears to be justified. A poll conducted in early September 2022 indicates that other Republicans and the general public may share this concern [40]. According to media reports, 58% of respondents, and approximately 25% of Republicans, “said Trump’s ‘Make America Great Again’ movement is threatening America’s democratic foundations.” Most Republicans (60%) did not think that “Trump’s MAGA movement represents the majority of the party.” Analyses of the 2022 US midterm elections suggest that these concerns among Republicans contributed to the defeat of candidates who had denied Trump’s loss in the 2020 presidential elections [41, 42].

Our findings provide grounds for guarded optimism and a specific point of focus for potential interventions to reduce the risk of violence. In this analysis, despite their increased endorsement of political violence, MAGA Republicans were not more willing than others to engage in violence themselves. This is concordant with findings in prior reports from our survey, in which the vast majority of those who saw political violence as usually or always justified were unwilling to participate in such violence [6, 8, 9]. Optimism here should be tempered by the knowledge that widespread support for political violence, even when most supporters are not personally willing to engage in violence, can still incite violence by those who are willing.

Fortunately, new research is identifying potentially efficacious interventions for adaptation to specific high-risk groups or implementation at the population level [1, 2, 43, 44]. Given our findings, intervention experts should consider how best to prevent a transition among MAGA Republicans from support for political violence to willingness to engage in it. More research is needed on the factors that lead to support for political violence; addressing those factors is a principal strategy for lessening such support in the future [1, 2].

### Limitations

Several technical limitations exist and were reported previously [6, 8, 9]; they are restated here. The findings are cross-sectional and subject to sampling error and bias due to nonresponse and other factors. Crosstabulations frequently produce response counts <100, and weighted prevalences for some important outcomes are below 5%. The large study sample results in relatively narrow confidence intervals in these cases, and sensitivity analysis indicated results were robust to the exclusion of respondents who failed attentiveness checks. Widely publicized mass shootings occurred in Buffalo, NY and Uvalde, TX while the survey was in the field. The Buffalo shooting is understood to have been a race-related hate crime motivated by “great replacement” thinking and may have affected respondents’ views on race, violence, and that particular belief. Russia’s war against Ukraine may have influenced responses on violence and democracy.

Other limitations are specific to this analysis. Our violence outcomes are all markers for potential risk. Acts of political violence would be a stronger outcome measure, and data on acts of violence would also be necessary to validate the measures used here. Such data are not available. To the extent that respondents viewed personal willingness to engage in violence as stigmatized behavior, our results may reflect under-reporting. We note, however, that these questions were asked only of respondents who considered political violence to be justified.

## Conclusion

Findings from this large, nationally representative survey demonstrate that, as defined, MAGA Republicans are more likely than others to endorse political violence and differ significantly from other Republicans on many measures. Concern about the potential for political violence among MAGA Republicans appears to be justified, but it is noteworthy that they were not more willing than others to engage in violence themselves. Further research on factors associated with willingness to commit political violence is urgently needed to support effective prevention measures.

## Supporting information

S1 File The supplement contains questions that supplied data for this analysis, references for those questions, and the following 10 supplementary tables. (DOCX)

S1 Data Data zip files included tables S1-S11. (ZIP)
