# Supplementary material for: MAGA Republicans’ views of American democracy and society and support for political violence in the United States: Findings from a nationwide population-representative survey
Source: PLoS One. 2024 Jan 3;19(1):e0295747. doi: 10.1371/journal.pone.0295747 (PMC10763974; doi:10.1371/journal.pone.0295747)
Supplement: S1 File — (DOCX) [file pone.0295747.s001.docx]

Supplement

MAGA Republicans’ views of American democracy and society and support for political violence in the United States: findings from a nationwide population-representative survey

Garen J. Wintemute, MD, MPH; Sonia L. Robinson, PhD, MPH; Elizabeth A. Tomsich, PhD; Daniel J. Tancredi, PhD

This supplement has been provided by the authors to give readers additional information about the work.

| **Page** | **Item** |
| --- | --- |
| 2 | Questions from the KnowledgePanel profile questionnaire that supplied data for this study |
| 3 | Questions from the 2022 American Life Survey that supplied data for this study |
| 9 | References for the question list |
| 10 | S1 Table. Prevalence differences for beliefs concerning democracy in the US, by study group |
| 12 | S2 Table. Prevalence differences for beliefs concerning the potential need for violence in the US (strongly or very strongly agree), by study group |
| 13 | S3 Table. Prevalence differences for beliefs concerning race and ethnicity and American society (strongly or very strongly agree), by study group |
| 14 | S4 Table. Prevalence differences for beliefs concerning QAnon and biblical “end times” (strongly or very strongly agree), by study group |
| 15 | S5 Table. Prevalence differences for justification for violence in non-political situations (usually or always justified), by study group |
| 16 | S6 Table. Prevalence differences for justification for political violence “in general” and to advance 9 specific political objectives (usually or always justified), by study group |
| 18 | S7 Table. Prevalence differences for justification for political violence to advance 8 additional specific objectives (usually or always justified), by study group (these objectives were paired, with respondents randomized 1:1 to see 1 item in each pair) |
| 20 | S8 Table. Prevalence differences for personal willingness to engage in political violence (very or completely willing), by type of violence and study group |
| 21 | S9 Table. Prevalence differences for personal willingness to engage in political violence (very or completely willing), by target of violence and study group |
| 23 | S10 Table. Prevalence differences for future likelihood of firearm possession and use in a situation where political violence is perceived as justified (very or extremely likely), by study group |

**Questions from the KnowledgePanel profile questionnaire that supplied data for this study**

**Political ideology**

**Q:** In general, do you think of yourself as...

1. Extremely liberal

2. Liberal

3. Slightly liberal

4. Moderate/middle of the road

5. Slightly conservative

6. Conservative

7. Extremely conservative

**Voting behavior**

**Q:** Did you happen to vote in the November 2020 elections for the U.S. President and Congress?

1. Yes

2. No

**Q:** Which candidate did you vote for in the 2020 Presidential election?

1. Joe Biden (Democrat)

2. Donald Trump (Republican)

3. Another candidate, please specify

**Questions from the 2022 American Life Survey that supplied data for this study**

For questions that presented a series of items for separate consideration, such as the third question below, all those items are listed, even though not all were used in this analysis.

Response options are presented here in order from negative to positive (e.g., “not important” to “extremely important”). Respondents were randomized 1:1 to receive responses in that order or the reverse.

In the list below, questions or items that were repeated or adapted from prior surveys contain citations to those surveys.

**Domain 1: democracy in the United States**

Now we’d like to ask you a few questions about the United States as you see it now, in 2022.

**Q:** Do you believe that things in this country today are…?[1]

1. Generally headed in the wrong direction

2. Generally headed in the right direction

**Q:** When thinking about democracy in the United States these days, do you believe…?[2]

1. There is a serious threat to our democracy.

2. There may be a threat to our democracy, but it is not serious.

3. There is no threat to our democracy.

**Q:** How important do you think it is for the United States to remain a democracy?[3]

1. Not important

2. Somewhat important

3. Very important

4. Extremely important

**Q:** How much do you agree or disagree with the following statements about democracy in the United States?

a. Democracy is the best form of government.[4]

b. These days, American democracy only serves the interests of the wealthy and powerful.[4]

c. Having a strong leader for America is more important than having a democracy.

d. If elected leaders will not protect American democracy, the people must do it themselves, even if it requires taking violent actions.[4]

e. In the next few years, there will be civil war in the United States.[5]

1. Do not agree

2. Somewhat agree

3. Strongly agree

4. Very strongly agree

**Domain 2: American society and institutions**

The next few questions are about your views of American society.

**Q:** How much do you agree or disagree with each of the following statements about people in America today?

a. White people benefit from advantages in society that Black people do not have.[4]

b. Discrimination against whites is as big a problem as discrimination against Blacks and other minorities.[4]

c. Our American way of life is disappearing so fast that we may have to use force to save it.[4]

d. In America, native-born white people are being replaced by immigrants.

e. Having more Black Americans, Latinos, and Asian Americans is good for the country.[6]

1. Do not agree

2. Somewhat agree

3. Strongly agree

4. Very strongly agree

**Q:** People have many different views about American society. How much do you agree or disagree with each of the following?

a. The government, media, and financial worlds in the U.S. are controlled by a group of Satan-worshipping pedophiles who run a global child sex trafficking operation.[7]

b. There is a storm coming soon that will sweep away the elites in power and restore the rightful leaders.[7]

c. Because things have gotten so far off track, true American patriots may have to resort to violence in order to save our country.[7]

d. The chaos in America today is evidence that we are living in what the Bible calls “the end times.”[8]

e. Capitalism is a system of oppression and should be abolished.

f. Straight white men hold far too much power in America.

g. The 2020 election was stolen from Donald Trump, and Joe Biden is an illegitimate president.

h. Armed citizens should patrol polling places at election time.

1. Do not agree

2. Somewhat agree

3. Strongly agree

4. Very strongly agree

**Domain 3: violence, including political violence**

Now we have a few questions about the use of force or violence. A reminder: your responses will be kept confidential and anonymous.

**Q:** In general, what do you think about the use of force or violence in the following situations—is it never justified, sometimes justified, usually justified, or always justified? “Force or violence” means physical force strong enough that it could cause pain or injury to a person.

(Not randomized)

a. In self defense

b. To prevent someone from injuring or killing another person

c. To prevent someone from injuring or killing themselves

d. To prevent harm or damage to property

e. To win an argument

f. In response to an insult

g. To get respect

1. Never justified

2. Sometimes justified

3. Usually justified

4. Always justified

**Q:** People sometimes talk about using force or violence to achieve political objectives. In general, what do you think about using force or violence to advance an important political objective that you support—is it…?

1. Never justified

2. Sometimes justified

3. Usually justified

4. Always justified

**Q:** Again, your view of the use of force or violence to advance an important political objective might depend on the specific objective that was involved. What do you think about the use of force or violence in the following situations—is it never justified, sometimes justified, usually justified, or always justified?

a. To return Donald Trump to the presidency this year

b. To stop an election from being stolen

c. To stop people who do not share my beliefs from voting

d. To prevent discrimination based on race or ethnicity

e. To preserve an American way of life based on Western European traditions

f. To oppose the government when it does not share my beliefs

g. To oppose the government when it tries to take private land for public purposes

1. Never justified

2. Sometimes justified

3. Usually justified

4. Always justified

The next questions are about your personal willingness to use force or violence.

**(Questions asked of respondents who endorsed at least 1 use of violence to achieve a specific political objective.)**

**Q:** In a situation where you think force or violence is justified to advance an important political objective, how willing would you personally be to use force or violence in each of these ways?

a. To damage property

b. To threaten or intimidate a person

c. To injure a person

d. To kill a person

1. Not willing

2. Somewhat willing

3. Very willing

4. Completely willing

**Q:** In a situation where you think force or violence is justified to advance an important political objective, how willing would you personally be to use force or violence against a person because they are…

a. An elected federal or state government official

b. An elected local government official

c. A public health official

d. A member of the military or National Guard

e. A police officer

f. A person who does not share your race or ethnicity

g. A person who does not share your religion

h. An election worker, such as a poll worker or vote counter

i. A person who does not share your political beliefs

1. Not willing

2. Somewhat willing

3. Very willing

4. Completely willing

**(Question asked of all respondents.)**

**Q:** Thinking now about the future and all the changes it might bring, how likely is it that you will use a gun in any of the following ways in the next few years—in a situation where you think force or violence is justified to advance an important political objective?

a. I will be armed with a gun.

b. I will carry a gun openly, so that people know I am armed.

c. I will threaten someone with a gun.

d. I will shoot someone with a gun.

1. Not likely

2. Somewhat likely

3. Very likely

4. Extremely likely

**References for the question list**

1. The Economist/YouGov Poll. 2021 June 13-16. https://docs.cdn.yougov.com/uagnfc262c/econTabReport.pdf.NPR/PBS

2. NewsHour/Marist National Poll. Trust in elections, threat to democracy, November 2021. 2021 November 1. https://maristpoll.marist.edu/polls/npr-pbs-newshour-marist-national-poll-trust-in-elections-threat-to-democracy-biden-approval-november-2021/.

3. Grinnell College National Poll. 52% of Americans believe democracy facing “major threat.” Study #2243. 2021 October 20. https://www.grinnell.edu/news/52-americans-believe-democracy-facing-major-threat.

4. Survey Center on American Life. January 2021 American Perspectives Survey topline questionnaire. https://www.americansurveycenter.org/wp-content/uploads/2021/03/January-2021-APS-Topline-Questionnaire.pdf.

5. Zogby. Will the US have another civil war? 2021 Feb 4. https://zogbyanalytics.com/news/997-the-zogby-poll-will-the-us-have-another-civil-war

6. Pew Research Center. Americans see advantages and challenges in country’s growing racial and ethnic diversity. 2019 May. https://www.pewresearch.org/social-trends/2019/05/08/americans-see-advantages-and-challenges-in-countrys-growing-racial-and-ethnic-diversity/.

7. Public Religion Research Institute. The persistence of Q-Anon in the post-Trump era: an analysis of who believes the conspiracies. 2022 Feb 24. https://www.prri.org/research/the-persistence-of-qanon-in-the-post-trump-era-an-analysis-of-who-believes-the-conspiracies/.

8. IFYC – PRRI Survey on Religion & COVID-19 Vaccine Trust. 2021 March. https://www.prri.org/wp-content/uploads/2021/05/Topline-IFYC-PRRI-Survey-on-Religion-and-COVID-19-Vaccine-Trust-v2_final.pdf.

S1 Table. Prevalence differences for beliefs concerning democracy in the US, by study group

Model 0 is unadjusted; Model 1 includes age, gender, and race and ethnicity; Model 2 includes those variables and income, education, and Census region. Prevalence differences and their 95% CIs are from a linear regression model for complex surveys where the dependent variable was coded as a dichotomous response.

* Denotes self-identified Republicans who voted for Donald Trump in 2020 and agreed strongly or very strongly with the statement that “the 2020 election was stolen from Donald Trump, and Joe Biden is an illegitimate president.”

S2 Table. Prevalence differences for beliefs concerning the potential need for violence in the US (strongly or very strongly agree), by study group

Model 0 is unadjusted; Model 1 includes age, gender, and race and ethnicity; Model 2 includes those variables and income, education, and census division (see footnote to Table 1 for definitions of census divisions). Prevalence differences and their 95% CIs are from a linear regression model for complex surveys where the dependent variable was coded as a dichotomous response.

* Denotes self-identified Republicans who voted for Donald Trump in 2020 and agreed strongly or very strongly with the statement that “the 2020 election was stolen from Donald Trump, and Joe Biden is an illegitimate president.”

S3 Table. Prevalence differences for beliefs concerning race and ethnicity and American society (strongly or very strongly agree), by study group

Model 0 is unadjusted; Model 1 includes age, gender, and race and ethnicity; Model 2 includes those variables and income, education, and census division (see footnote to Table 1 for definitions of census divisions). Prevalence differences and their 95% CIs are from a linear regression model for complex surveys where the dependent variable was coded as a dichotomous response.

* Denotes self-identified Republicans who voted for Donald Trump in 2020 and agreed strongly or very strongly with the statement that “the 2020 election was stolen from Donald Trump, and Joe Biden is an illegitimate president.”

S4 Table. Prevalence differences for beliefs concerning QAnon and biblical “end times” (strongly or very strongly agree), by study group

Model 0 is unadjusted; Model 1 includes age, gender, and race and ethnicity; Model 2 includes those variables and income, education, and census division (see footnote to Table 1 for definitions of census divisions). Prevalence differences and their 95% CIs are from a linear regression model for complex surveys where the dependent variable was coded as a dichotomous response.

* Denotes self-identified Republicans who voted for Donald Trump in 2020 and agreed strongly or very strongly with the statement that “the 2020 election was stolen from Donald Trump, and Joe Biden is an illegitimate president.”

S5 Table. Prevalence differences for justification for violence in non-political situations (usually or always justified), by study group

Model 0 is unadjusted; Model 1 includes age, gender, and race and ethnicity; Model 2 includes those variables and income, education, census division (see footnote to Table 1 for definitions of census divisions). Prevalence differences and their 95% CIs are from a linear regression model for complex surveys where the dependent variable was coded as a dichotomous response.

* Denotes self-identified Republicans who voted for Donald Trump in 2020 and agreed strongly or very strongly with the statement that “the 2020 election was stolen from Donald Trump, and Joe Biden is an illegitimate president.”

S6 Table. Prevalence differences for justification for political violence “in general” and to advance 9 specific political objectives (usually or always justified), by study group

Model 0 is unadjusted; Model 1 includes age, gender, and race and ethnicity; Model 2 includes those variables and income, education, and census division (see footnote to Table 1 for definitions of census divisions). Prevalence differences and their 95% CIs are from a linear regression model for complex surveys where the dependent variable was coded as a dichotomous response.

* Denotes self-identified Republicans who voted for Donald Trump in 2020 and agreed strongly or very strongly with the statement that “the 2020 election was stolen from Donald Trump, and Joe Biden is an illegitimate president.”

S7 Table. Prevalence differences for justification for political violence to advance 8 additional specific objectives (usually or always justified), by study group (these objectives were paired, with respondents randomized 1:1 to see 1 item in each pair)

Model 0 is unadjusted; Model 1 includes age, gender, and race and ethnicity; Model 2 includes those variables and income, education, and census division (see footnote to Table 1 for definitions of census divisions). Prevalence differences and their 95% CIs are from a linear regression model for complex surveys where the dependent variable was coded as a dichotomous response.

* Denotes self-identified Republicans who voted for Donald Trump in 2020 and agreed strongly or very strongly with the statement that “the 2020 election was stolen from Donald Trump, and Joe Biden is an illegitimate president.”

S8 Table. Prevalence differences for personal willingness to engage in political violence (very or completely willing), by type of violence and study group

Model 0 is unadjusted; Model 1 includes age, gender, and race and ethnicity; Model 2 includes those variables and income, education, and census division (see footnote to Table 1 for definitions of census divisions). Prevalence differences and their 95% CIs are from a linear regression model for complex surveys where the dependent variable was coded as a dichotomous response.

* Denotes self-identified Republicans who voted for Donald Trump in 2020 and agreed strongly or very strongly with the statement that “the 2020 election was stolen from Donald Trump, and Joe Biden is an illegitimate president.”

S9 Table. Prevalence differences for personal willingness to engage in political violence (usually or always justified), by target of violence and study group

Model 0 is unadjusted; Model 1 includes age, gender, and race and ethnicity; Model 2 includes those variables and income, education, and census division (see footnote to Table 1 for definitions of census divisions). Prevalence differences and their 95% CIs are from a linear regression model for complex surveys where the dependent variable was coded as a dichotomous response.

* Denotes self-identified Republicans who voted for Donald Trump in 2020 and agreed strongly or very strongly with the statement that “the 2020 election was stolen from Donald Trump, and Joe Biden is an illegitimate president.”

S10 Table. Prevalence differences for future likelihood of firearm possession and use in a situation where political violence is perceived as justified (very or extremely likely), by study group

Model 0 is unadjusted; Model 1 includes age, gender, and race and ethnicity; Model 2 includes those variables and income, education, and census division (see footnote to Table 1 for definitions of census divisions). Prevalence differences and their 95% CIs are from a linear regression model for complex surveys where the dependent variable was coded as a dichotomous response.

* Denotes self-identified Republicans who voted for Donald Trump in 2020 and agreed strongly or very strongly with the statement that “the 2020 election was stolen from Donald Trump, and Joe Biden is an illegitimate president.”
